# Standardisation of oxygen exposure in the development of mouse models for bronchopulmonary dysplasia

**DOI:** 10.1242/dmm.027086

**Published:** 2017-02-01

**Authors:** Claudio Nardiello, Ivana Mižíková, Diogo M. Silva, Jordi Ruiz-Camp, Konstantin Mayer, István Vadász, Susanne Herold, Werner Seeger, Rory E. Morty

**Affiliations:** 1 Department of Lung Development and Remodelling, Max Planck Institute for Heart and Lung Research, 61231 Bad Nauheim, Germany; 2 Department of Internal Medicine (Pulmonology), University of Giessen and Marburg Lung Center (UGMLC), member of the German Center for Lung Research (DZL), 35392 Giessen, Germany

**Keywords:** BPD, Hyperoxia, Alveolarisation, Structure, Animal model

## Abstract

Progress in developing new therapies for bronchopulmonary dysplasia (BPD) is sometimes complicated by the lack of a standardised animal model. Our objective was to develop a robust hyperoxia-based mouse model of BPD that recapitulated the pathological perturbations to lung structure noted in infants with BPD. Newborn mouse pups were exposed to a varying fraction of oxygen in the inspired air (FiO_2_) and a varying window of hyperoxia exposure, after which lung structure was assessed by design-based stereology with systemic uniform random sampling. The efficacy of a candidate therapeutic intervention using parenteral nutrition was evaluated to demonstrate the utility of the standardised BPD model for drug discovery. An FiO_2_ of 0.85 for the first 14 days of life decreased total alveoli number and concomitantly increased alveolar septal wall thickness, which are two key histopathological characteristics of BPD. A reduction in FiO_2_ to 0.60 or 0.40 also caused a decrease in the total alveoli number, but the septal wall thickness was not impacted. Neither a decreasing oxygen gradient (from FiO_2_ 0.85 to 0.21 over the first 14 days of life) nor an oscillation in FiO_2_ (between 0.85 and 0.40 on a 24 h:24 h cycle) had an appreciable impact on lung development. The risk of missing beneficial effects of therapeutic interventions at FiO_2_ 0.85, using parenteral nutrition as an intervention in the model, was also noted, highlighting the utility of lower FiO_2_ in selected studies, and underscoring the need to tailor the model employed to the experimental intervention. Thus, a state-of-the-art BPD animal model that recapitulates the two histopathological hallmark perturbations to lung architecture associated with BPD is described. The model presented here, where injurious stimuli have been systematically evaluated, provides a most promising approach for the development of new strategies to drive postnatal lung maturation in affected infants.

## Abstract (precis)

**Summary:** A newborn mouse model of bronchopulmonary dysplasia in which oxygen injury is systematically standardised recapitulates the two pathological hallmarks of disease.

## INTRODUCTION

Precise modelling of human disease using animal models is a major challenge in translating bench science to the bedside, as (1) animal models must accurately recapitulate disease processes to facilitate the identification of pathogenic pathways, and (2) animal models represent the limiting step in assessing which therapeutic interventions hold promise for subsequent study. This is particularly evident in animal models of human diseases that are characterised by perturbations to the architecture of an organ. Modelling disease pathogenesis in experimental animals is problematic from multiple perspectives. Amongst these, the injurious insult employed in the experimental model might not recapitulate key elements of disease, thereby limiting the ability to evaluate the efficacy of candidate therapeutic agents. Furthermore, the precision of the readout that is employed might be inadequate to detect small changes in anatomical structures that are targeted by both the injurious insult and candidate therapeutic intervention. A further confounding variable is the use of experimental animals in medical research, as emphasis must be placed on ‘reduction, refinement and replacement’ (the 3R concept) (Curzer et al., 2016), where the number of experimental animals employed and the level of stress to which the animal is subjected must be maintained at the minimum level possible, while still retaining the translational viability of the animal model.

Modelling bronchopulmonary dysplasia (BPD) in experimental animals is a textbook illustration of these concerns. BPD is the most common complication of preterm birth and represents significant morbidity and mortality in the neonatal intensive care unit (Jobe, 2011; Jobe and Tibboel, 2014). BPD is caused by a combination of the toxic effects of oxygen supplementation used to manage respiratory failure in preterm infants, baro- and volu-trauma from mechanical ventilation (Greenough et al., 2008), as well as other disease-modifying variables such as infection and inflammation (Balany and Bhandari, 2015). BPD results in long-term complications in respiratory function that persist into adulthood (Hilgendorff and O'Reilly, 2015). The pathology of BPD has changed over time; where ‘old’ BPD, which is particularly characterised by fibrosis, thickened septa and some alveolar simplification, results from aggressive mechanical ventilation with high oxygen levels. In contrast, ‘new’ BPD, which is the prevalent form today, is largely characterised by alveolar simplification, where preterm infants are less aggressively ventilated, with lower oxygen levels (Jobe, 2011). As a result of improvements in the medical management of BPD, the incidence of BPD is increasing because preterm infants delivered earlier have increasingly improved survival (Stoll et al., 2015). This underscores a pressing need to develop new medical management strategies. However, these efforts might be hampered by the lack of appropriate animal models of BPD. In affected individuals, two key elements of the lung structure are impacted: the oxygen supplementation arrests lung development, which causes fewer alveoli of a larger size to be generated. Concomitantly, the thickness of the delicate barrier between the alveolar airspaces in the lung and the capillary network of the lung is thickened, which compromises gas exchange, and is evident by thickened alveolar septal walls. In order to properly model BPD, the injurious intervention (oxygen supplementation) must recapitulate both structural elements of the pathology, a decreased number of alveoli and an increased alveolar septal wall thickness.

To this end, a large number of methodologies have been reported that rely on the exposure of newborn mouse pups to an increased fraction of oxygen in the inspired air (FiO_2_), often reported as a percentage of oxygen in the inspired air, which ranges between 40% O_2_ (FiO_2_ 0.4) and 100% O_2_ (FiO_2_ 1.0) (Silva et al., 2015). Between 1 January 2013 and 30 June 2015, 41 different oxygen exposure protocols had been reported in BPD animal models (Silva et al., 2015), sometimes with diametrically opposite findings concerning the effects of the same intervention in different animal models of BPD (for example, compare the studies of Britt et al., 2013 and Masood et al., 2014). This underscores the impact of the oxygen exposure regimen selected on data analysis and study conclusions. Of course, it must be acknowledged that diversity in animal models is also a strength of the BPD field, where BPD has a multifactorial aetiology that cannot be fully modelled in a single, standardised animal model.

The analysis of lung structure in material from animal models of BPD is also problematic (Silva et al., 2015). Currently, a mixture of approaches is employed, often based on the analysis of paraffin-embedded lung tissue with classical determinants of mean linear intercept (MLI) and radial alveolar count (RAC) as surrogates of the size of the alveoli (Silva et al., 2015). These determinations are made by direct measurement of the distance between the adjacent walls of an alveolus. Similarly, the thickness of the alveolar wall is directly assessed using a slide-rule and visual inspection, which is not unbiased. Furthermore, the highly elastic structure of the lung results in substantial distortion of the lung structure during the dehydration and rehydration of the lung during paraffin embedding (Schneider and Ochs, 2014). These are all important concerns in the analysis of the lung architecture, although these approaches have been successfully used to identify potentially important pathogenic pathways and for the pre-clinical evaluation of candidate therapeutic interventions (Liao et al., 2015; Olave et al., 2016; Tibboel et al., 2013).

To address the concerns outlined above, important advances have been made replacing paraffin embedding with plastic embedding, along with treatment of lung tissue with arsenic, osmium, and uranium, which results in appreciable preservation of the lung structure (Schneider and Ochs, 2014). Furthermore, a design-based stereological approach to the analysis of organ structure has been developed, and continues to be refined (Schneider and Ochs, 2013; Tschanz et al., 2014). This approach represents a substantial advance over the MLI and RAC methods when applied to the lung, as the technique is both unbiased (thus not subjective), and has a very high resolution (Mühlfeld and Ochs, 2013; Ochs and Mühlfeld, 2013). Design-based stereology has recently been applied to the analysis of the architecture of developing lungs of newborn mouse pups by the authors, for the first time allowing the assessment of the total number of alveoli in the lung, and the assessment of changes <1 µm in magnitude during perturbations to newborn mouse lung development (Madurga et al., 2015, 2014; Mižíková et al., 2015).

These recent developments in tissue embedding and structural analysis represent an important advance in our ability to study lung development. Here, we employed state-of-the-art tissue embedding methodology, together with state-of-the-art design-based stereology, to identify the magnitude of oxygen toxicity and the window of oxygen exposure that is required to model BPD as perfectly as possible in mice. The primary threshold parameters selected were a decrease in total alveoli number in the lung, and an increase in the alveolar septal wall thickness, which are the two anatomical hallmarks of BPD. We then documented the extraordinary impact of using different oxygen levels in the experimental model on the observed efficacy of a candidate intervention, thus highlighting the crucial importance of selecting the correct model to evaluate experimental therapeutics.

## RESULTS

**Fig. 1.**
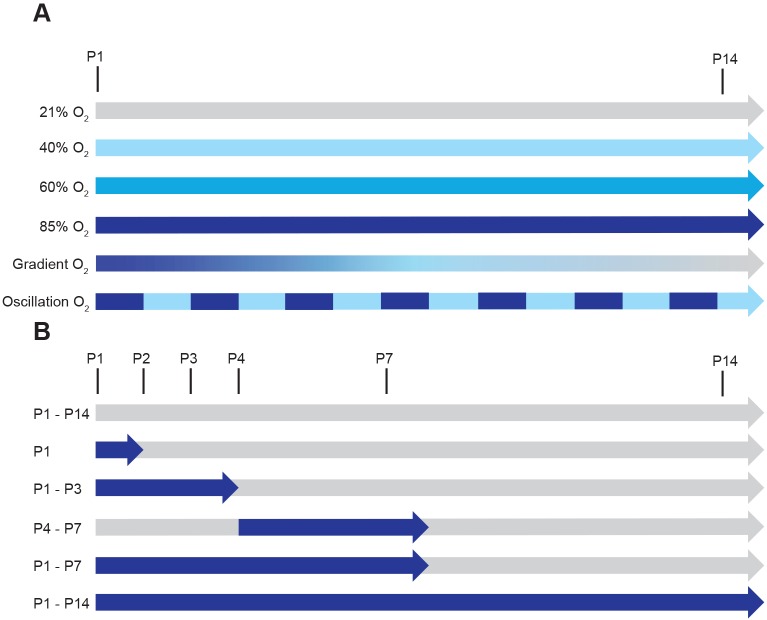
**Schematic illustration of the different oxygen exposure protocols employed.** (A) Determination of the effects of the level of oxygen employed as an injurious stimulus. Exposure protocols include the continuous exposure of newborn mouse pups from postnatal day (P)1 (the day of birth) to the end of P14, to ambient room air (21% O_2_), 40% O_2_, 60% O_2_, and 85% O_2_. Alternatively, newborn mouse pups were exposed to a decreasing gradient of O_2_ concentration from 85% O_2_ at P1 to 21% O_2_ at P14; or to an oscillation between 85% O_2_ and 40% O_2_ in an oscillation cycle of 24 h: 24 h, starting with 85% O_2_ on P1, and ending with 40% O_2_ on P14. (B) Determination of the effects of the time frame (window) of exposure to injurious oxygen levels. The exposure protocols included the continuous exposure of newborn mouse pups from P1 to the end of P14 to ambient room air (21% O_2_); to 85% O_2_ for the first 24 h of life, followed by 21% O_2_ from the start of P2 to the end of P14; to 85% O_2_ from P1 to the end of P3 followed by 21% O_2_ from the start of P4 to the end of P14; to 21% O_2_ from P1 to the end of P3, followed by 85% O_2_ from the start of P4 to the end of P7, followed by 21% O_2_ to the end of P14; to 85% O_2_ from P1 to the end of P7, followed by 21% O_2_ from the start of P8 to the end of P14; or to 85% O_2_ from P1 to the end of P14.

To identify the optimal injurious levels of oxygen, as well as the optimal window of exposure to injury, a series of oxygen exposure protocols were employed, which varied the FiO_2_ over the first 14 days of life (Fig. 1A) in a variety of windows of exposure (Fig. 1B).

### Identification of the optimal level of oxygen required to model BPD in mice

**Fig. 2.**
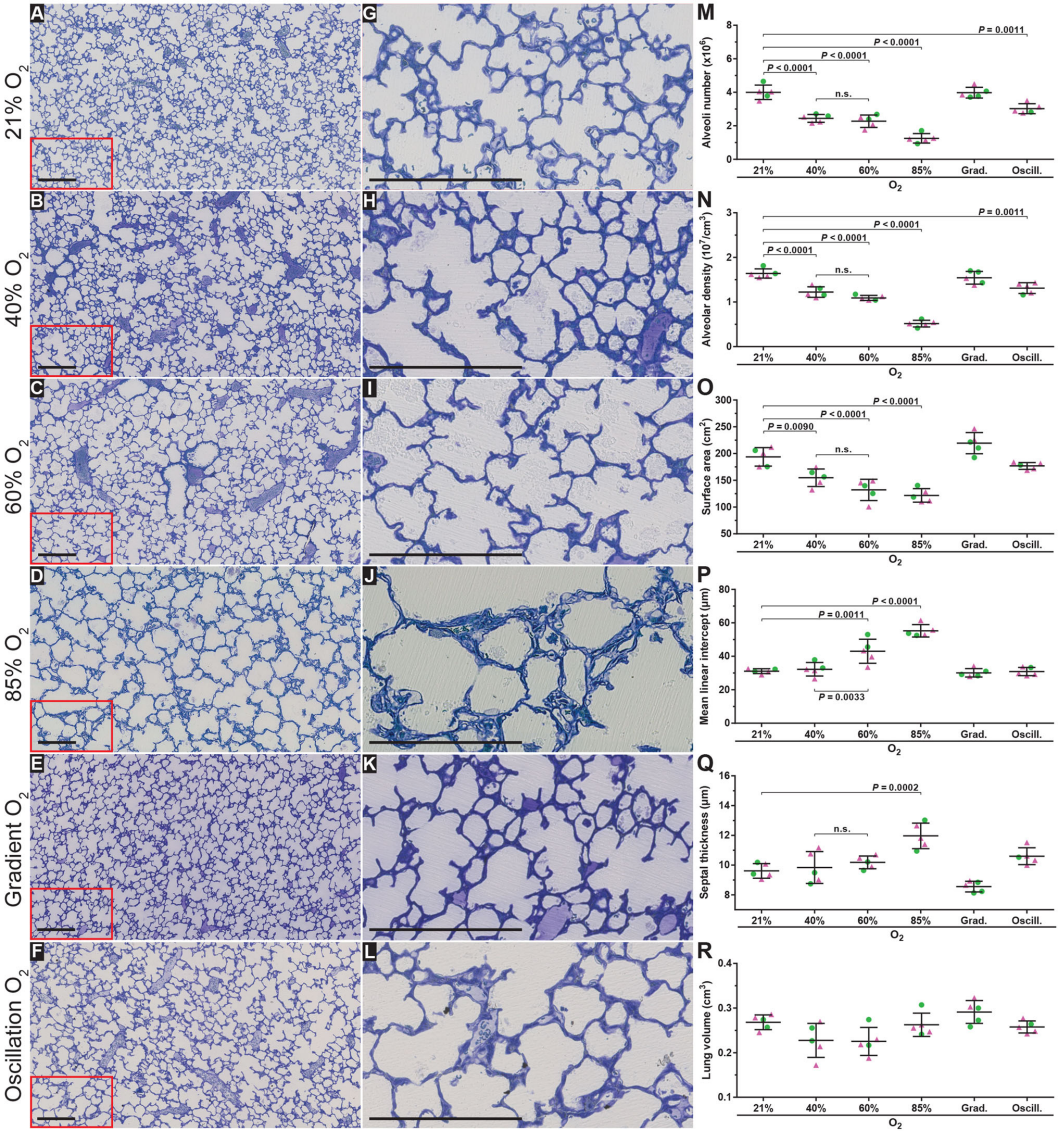
**Optimising oxygen exposure levels to model bronchopulmonary dysplasia in newborn mice.** Histological images were obtained from the lungs of mice subjected to the six oxygen exposure protocols illustrated in Fig. 1A. (A-F) Low-magnification images from the lungs, which were embedded in glycol methylacrylate plastic after fixation with buffered paraformaldehyde/glutaraldehyde and treatment with sodium cacodylate, osmium tetroxide, and uranyl acetate, then stained with Richardson's stain. (G-L) Higher-magnification images derived from part (demarcated by the red box) of the corresponding images to the left, to highlight changes in alveolar septal wall thickness. Each image is representative of lung sections obtained from four other mouse pups within each experimental group (*n*=5, per group). Scale bars: 200 µm. (M-Q) Design-based stereology was employed to assess (M) total number of alveoli in the lung, (N) alveolar density, (O) gas exchange surface area, (P) mean linear intercept, and (Q) alveolar septal wall thickness. (R) The lung volume was estimated by the Cavalieri method. In panels M-R, • denotes male animals, ▴ denotes female animals. Data represented as mean±s.d. Data comparisons were made by one-way ANOVA with Tukey's post hoc test. Significant *P*-values are indicated in the graphs; n.s., not significant (*P*≥0.05).

Fourteen days after birth [postnatal day (P)14], the bulk of postnatal lung alveolarisation has been completed (Burri, 2006). Thus, maintaining newborn mouse pups (*n*=5 per experimental group) under room-air (21% O_2_) conditions, starting on the day of birth, up to and including P14, served as the control protocol and generated mouse lungs that exhibited an anatomically normal alveolar structure (Fig. 2A,G). Increasing the concentration of oxygen in the inspired air to 40% O_2_ over the first 14 days of life generated a less organised lung parenchymal architecture (Fig. 2B,H versus Fig. 2A,G) and decreased the total number of alveoli in the lung by 39% (Fig. 2M) in comparison with the 21% O_2_ (control) group (complete data set in Table 1). This was accompanied by a concomitant 25% decrease in the alveolar density (Fig. 2N) and a 20% decrease in the gas exchange surface area (Fig. 2O), whereas no impact on alveolar septal wall thickness was noted (Fig. 2Q). Increasing the concentration of oxygen in the inspired air to 60% O_2_ over the first 14 days of life did not further impact the visual appearance of the lung structure (Fig. 2C,I), which exhibited a comparable number of alveoli, alveolar density, gas exchange surface area, and alveolar septal wall thickness to the 40% O_2_ group (Fig. 2M-O,Q,R); however, the mean MLI was increased in the 60% O_2_ group (Fig. 2P). In contrast, newborn mouse pups exposed to 85% O_2_ for the first 14 days of life exhibited further reductions of 69% in number of alveoli (Fig. 2M), 69% in alveolar density (Fig. 2N) and 37% in gas exchange surface area (Fig. 2O**)** compared with the 21% O_2_ control group. An increase in alveolar septal wall thickness of 25% in comparison with 21% O_2_ controls was also noted (Fig. 2Q). Thus, 85% O_2_ was the only oxygen concentration that impacted both total number of alveoli as well as alveolar septal wall thickness. For this reason, we performed two additional studies: the exposure of newborn mouse pups to a gradient of decreasing oxygen concentration from 85% O_2_ on the day of birth to 21% O_2_ on P14 (amounting to a step-wise decrease of 5% O_2_ per day), as well as an oscillation between 85% O_2_ and 40% O_2_ on a 24 h:24 h oscillating cycle (Fig. 1A).

**Table 1.**
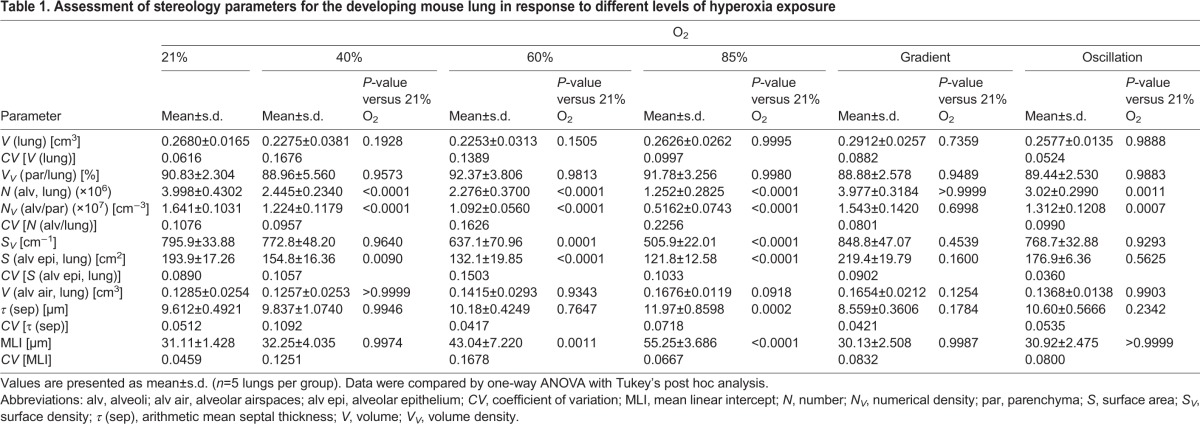
**Assessment of stereology parameters for the developing mouse lung in response to different levels of hyperoxia exposure**

The exposure of newborn mouse pups to a decreasing gradient of oxygen between 85% O_2_ and 21% O_2_ over the first 14 days of life had no impact on any parameter of lung architecture (Fig. 2E,K,M-R). In contrast, exposure of newborn mouse pups to an oscillation of 85% O_2_ and 40% O_2_ over the first 14 days of life generated lungs that were impacted on visual inspection, albeit lightly (Fig. 2F,L); and which exhibited a comparatively moderate reduction of 25% in the total number of alveoli in the lung (Fig. 2M) and 20% reduction in alveolar density (Fig. 2N), which was insufficient to significantly impact the gas exchange surface area, which remained unchanged (Fig. 2O). Similarly, alveolar septal wall thickness (Fig. 2Q) was unaffected. Changes in the MLI parameter largely paralleled those noted in other lung structure parameters (Fig. 2P). It is important to note that the MLI reported here represents a stereologically determined mean MLI over the whole lung, and not the potentially biased MLI determined by visual inspection of selected lung sections, which is widely reported in the literature. No change in lung volume was noted at P14 comparing all experimental groups (Fig. 2R), and none of the parameters assessed exhibited any clustering on the basis of the sex of the animals (Fig. 2M-R).

Together, these data demonstrate that exposure of newborn mouse pups to as little as 40% O_2_ over the first 14 days of life could recapitulate the changes seen in lung alveolar number in individuals with BPD; however, only exposure to 85% O_2_ could also recapitulate the changes noted in lung alveolar septal wall thickness in individuals with BPD.

### Identification of the optimal window of oxygen exposure required to model BPD in mice

**Fig. 3.**
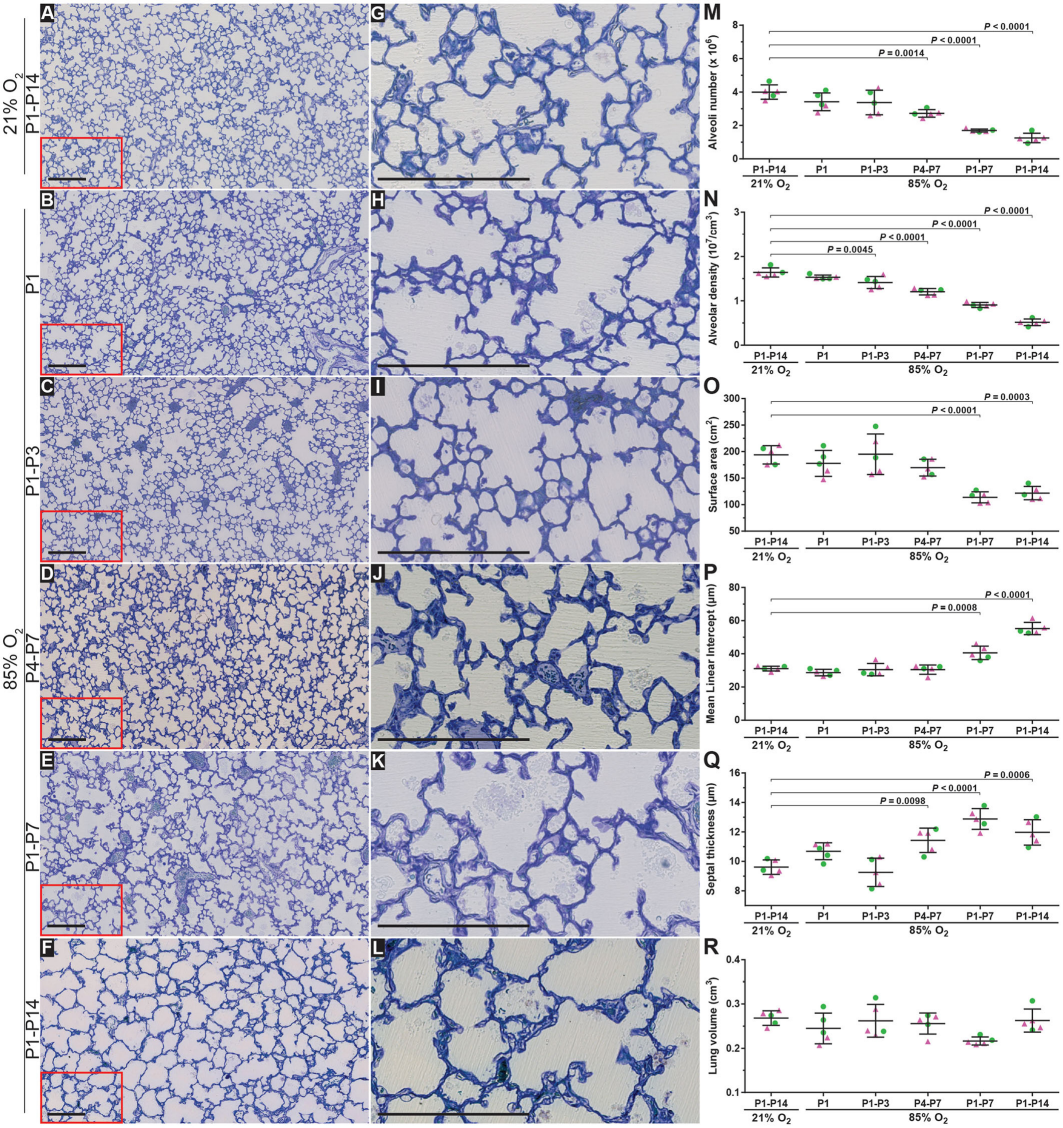
**Optimising the oxygen exposure window to model bronchopulmonary dysplasia in newborn mice.** Histological images were obtained from the lungs of mice subjected to the six oxygen exposure protocols illustrated in Fig. 1B. (A-F) Low-magnification images from the lungs, which were embedded in glycol methylacrylate plastic after fixation with buffered paraformaldehyde/glutaraldehyde and treatment with sodium cacodylate, osmium tetroxide, and uranyl acetate, then stained with Richardson's stain. (G-L) Higher-magnification images derived from part (demarcated by the red box) of the corresponding images to the left, to highlight changes in alveolar septal wall thickness. Each image is representative of lung sections obtained from four other mouse pups within each experimental group (*n*=5, per group). Scale bars: 200 µm. (M-Q) Design-based stereology was employed to assess (M) total number of alveoli in the lung, (N) alveolar density, (O) gas exchange surface area, (P) mean linear intercept, and (Q) alveolar septal wall thickness. (R) The lung volume was estimated by the Cavalieri method. In panels M-R, • denotes male animals, ▴ denotes female animals. Data represented as mean±s.d. Data comparisons were made by one-way ANOVA with Tukey's post hoc test. Significant *P*-values are indicated in the graphs.

Given that only exposure of newborn mouse pups to 85% O_2_ over the first 14 days of life could recapitulate the two hallmark perturbations to lung architecture seen in BPD, we then set out to assess the critical (and minimal) window of exposure to 85% O_2_, comparing five different windows of exposure (Fig. 1B) over the first 14 days of life (*n*=5 per experimental group). Exposure of newborn mouse pups to 85% O_2_ for the first 24 h of life only (followed by 13 days of 21% O_2_) had no impact on lung structure, either by visual inspection (Fig. 3B,H), or a design-based stereology analysis of the number of alveoli in the lung, the alveolar density, the gas exchange surface area, the MLI, or the alveolar septal wall thickness (Fig. 3M-R; complete data sets in Table 2). Increasing the window of exposure to include the first 72 h of life, from P1 up to and including P3, followed by 11 days of 21% O_2_ (Fig. 3C,I) generated a moderate (14%) decrease in alveolar density in comparison with the 21% O_2_ group (Fig. 3N), without impacting any other parameter. When an alternative 72 h window of exposure, where mouse pups were exposed to 85% O_2_ over a time-frame starting with and including P4, up to and including P7 was selected, a 32% decrease in the number of alveoli (Fig. 3M) and a 39% decrease in the alveolar density (Fig. 3N) in comparison with the 21% O_2_ group was noted. Although these changes were not sufficient to impact the gas exchange surface area (Fig. 3O), an increase in alveolar septal wall thickness of 19%, in comparison with 21% O_2_ was also noted (Fig. 3Q).

**Table 2.**
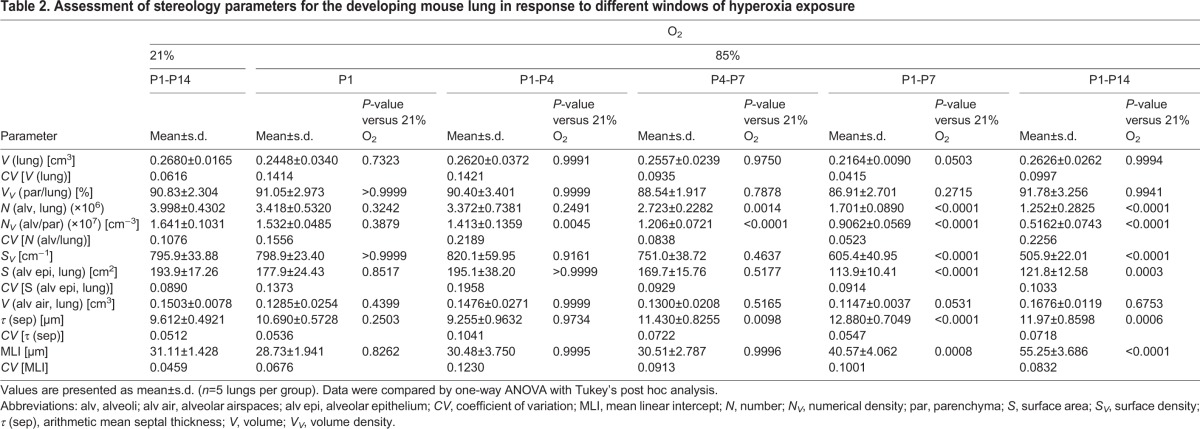
**Assessment of stereology parameters for the developing mouse lung in response to different windows of hyperoxia exposure**

Exposure of newborn mouse pups either to (1) 85% O_2_ from P1 to P7, followed by seven days of 21% O_2_ (Fig. 3E,K) or (2) to 85% O_2_ from P1 to P14 (Fig. 3F,L) generated the most dramatic impact on the lung architecture. A decrease in number of alveoli, alveolar density, and alveolar surface area, as well as an increase in MLI and alveolar septal wall thickness was noted in both groups (Fig. 3M-R), suggesting that these two protocols best model BPD in mice.

### Effect of oxygen exposure on the performance of a candidate therapeutic intervention

**Fig. 4.**
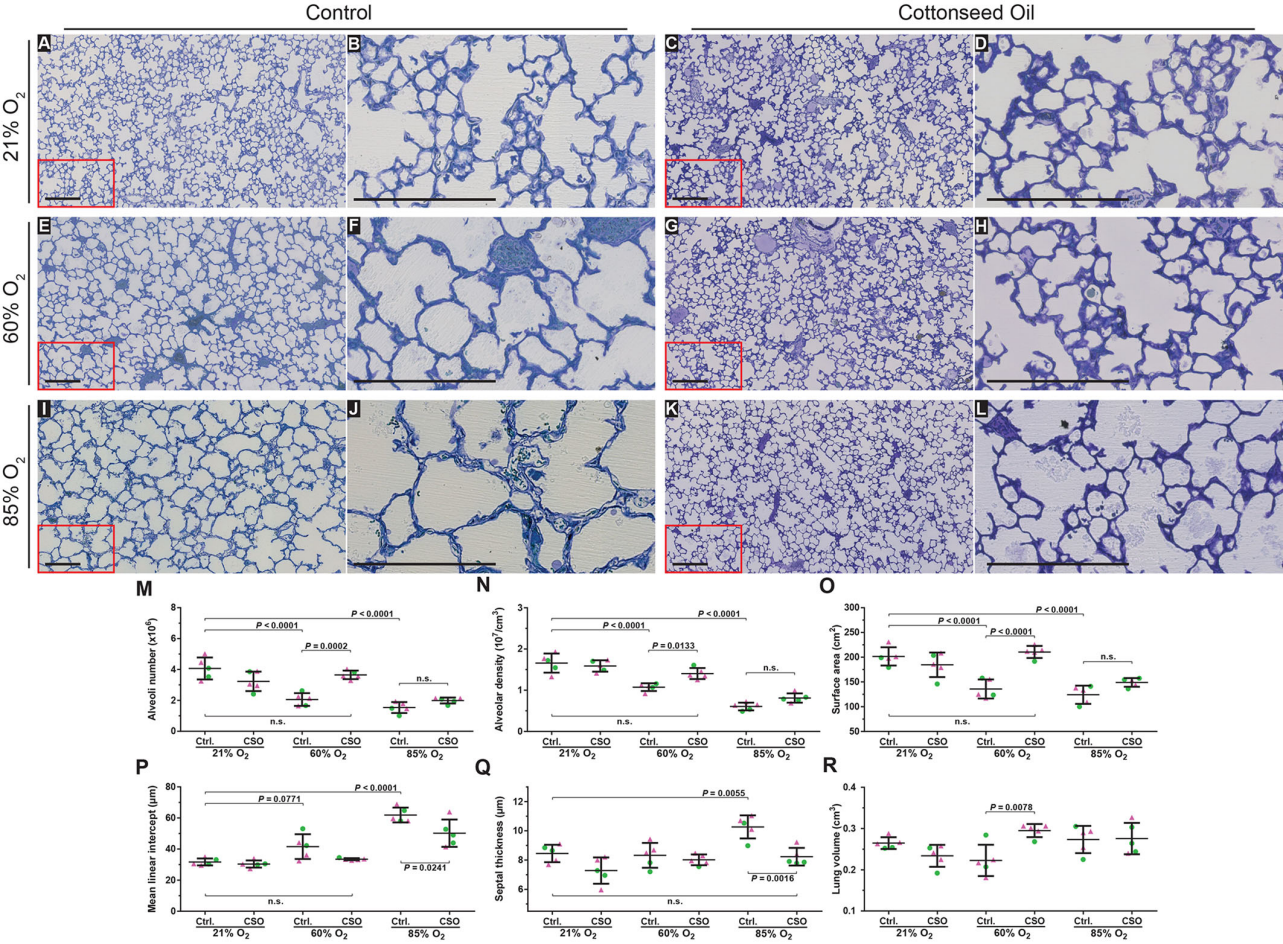
**Comparison of the efficacy of a candidate therapeutic intervention in a severe versus a less severe hyperoxia-based model of bronchopulmonary dysplasia.** Mouse pups within each of the three oxygen exposure groups [continuous ambient (21% O_2_) room air; 60% O_2_, or 85% O_2_; presented in rows] were either sham-treated (control) or treated with cottonseed oil (average of 12 ml kg^−1^ day^−1^; see Materials and Methods for precise dosing protocol) via daily intraperitoneal injection, to provide supplementary parenteral nutrition. (A-L) Images are sections from lungs embedded in glycol methylacrylate plastic after fixation with buffered paraformaldehyde/glutaraldehyde and treatment with sodium cacodylate, osmium tetroxide, and uranyl acetate, then stained with Richardson's stain. Each image is representative of lung sections obtained from four other mouse pups within each experimental group (*n*=5, per group). Within each treatment group (control versus cottonseed oil), the column of images to the left represents low-magnification images, with higher-magnification images derived from part (demarcated by the red box) of the corresponding images presented to the right, to highlight changes in alveolar septal wall thickness. Scale bars: 200 µm. (M-Q) Design-based stereology was employed to assess (M) total number of alveoli in the lung, (N) alveolar density, (O) gas exchange surface area, (P) mean linear intercept, and (Q) alveolar septal wall thickness. (R) The lung volume was estimated by the Cavalieri method. In panels G-L, • denotes male animals, ▴ denotes female animals. Data represented as mean±s.d. Data comparisons were made by one-way ANOVA with Tukey's post hoc test. Significant *P*-values are indicated in the graphs; n.s., not significant (*P*≥0.05). CSO, cottonseed oil-treated group; Ctrl., control (sham-treated) group.

Cottonseed oil was employed as an intervention for parenteral nutrition, and exhibited no impact on the structural development of newborn mouse lungs over the period P1 to P14 in mouse pups (*n*=5 per experimental group) maintained under 21% O_2_ compared with the lungs of control sham-treated mice maintained under 21% O_2_ (Fig. 4C,D versus Fig. 4A,B; Fig. 4M-R; complete data set in Table 3). Cottonseed oil tended to increase body mass (by up to 20%) and had no impact on survival (data not shown). When cottonseed oil was applied to newborn mouse pups exposed to 60% O_2_ over the period P1-P14, a pronounced recovery in the number of alveoli (an increase of 78%) was noted compared with sham-treated pups in the same oxygen-exposure protocol (Fig. 4G,H versus Fig. 4E,F; Fig. 4M). Similarly, cottonseed oil application increased the alveolar density by 31% (Fig. 4N) and increased the gas exchange surface area by 55% (Fig. 4O) in the 60% O_2_ group. These changes were accompanied by an increase in lung volume of 33% (Fig. 4R). As alveolar septal wall thickness was not impacted at 60% O_2_ (Fig. 2Q) no impact of cottonseed oil administration on alveolar septal wall thickness was expected, nor was any effect noted (Fig. 4Q). These data highlight the therapeutic benefit of cottonseed oil supplementation in this experimental animal model of BPD. However, when the more severe hyperoxia exposure model was employed with continuous exposure of newborn mouse pups to 85% O_2_ from P1 to P14, the beneficial impact of cottonseed oil administration on alveolar number and alveolar density was lost (Fig. 4K,L versus Fig. 4I,J; Fig. 4M,N). However, the impact of cottonseed oil on mean septal wall thickness was still noted (Fig. 4Q). Taken together, these data highlight a key concern of the 85% O_2_ exposure approach, where the extreme severity of the injurious stimulus might result in promising candidate therapeutic interventions being missed or discounted.

**Table 3.**
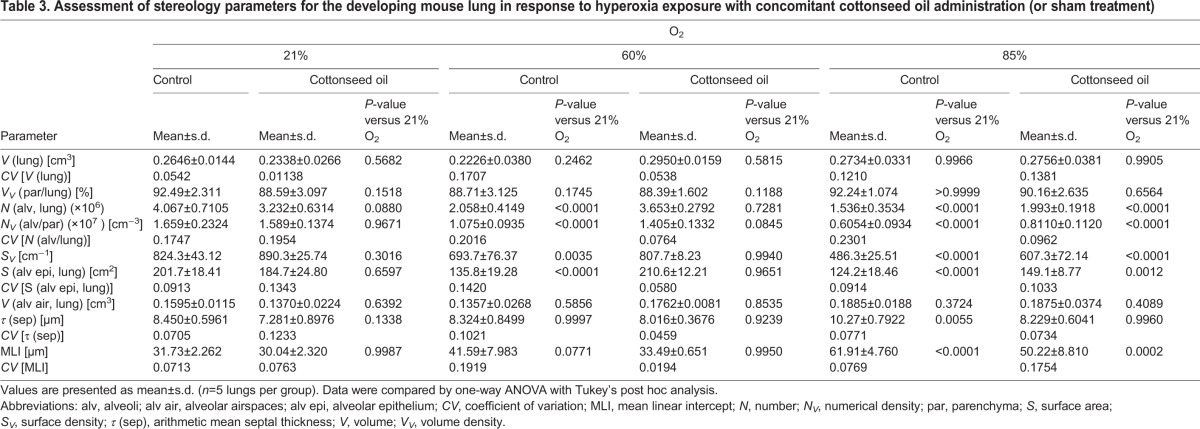
**Assessment of stereology parameters for the developing mouse lung in response to hyperoxia exposure with concomitant cottonseed oil administration (or sham treatment)**

## DISCUSSION

Given the increasing clinical burden of BPD (Jobe, 2011; Stoll et al., 2015), there is a pressing need for new therapeutic options for this syndrome. Progress in this regard has been impeded by a lack of suitable animal models, which represent the first step in the translational pipeline. Although nonhuman primates are a better laboratory model for these studies than are rodents (Herring et al., 2014), ethical considerations, and monetary and time costs support the use of rodents as first-line models for drug discovery and studies on disease pathogenesis. Thus, mice have found widespread application in BPD models (Berger and Bhandari, 2014), particularly because of the availability of transgenic mouse lines. Most mouse BPD models are based on exposure of newborn pups to elevated oxygen levels, as oxygen toxicity to the developing lung is the medical basis of BPD in preterm infants (Jobe, 2011; Stoll et al., 2015), and oxygen toxicity is known to disrupt elements of alveolar development including the maturation of the extracellular matrix (Mižíková and Morty, 2015), microRNA dynamics (Nardiello and Morty, 2016), as well as stem cell plasticity (Domm et al., 2015; Yee et al., 2016). However, there is a pronounced lack of standardisation of BPD mouse models, with over 41 different oxygen-exposure protocols reported in a 30-month period alone (Silva et al., 2015). Although initially very high (90-100%) oxygen concentrations were employed in the past as an injurious stimulus in BPD models, and indeed, still are by many groups; there has been a gradual shift in the degree of oxygen toxicity employed, with increased instances of oxygen concentrations below 80% O_2_ becoming increasingly evident in recent published studies (Bouch et al., 2016, 2015; Ehrhardt et al., 2016; Maduekwe et al., 2015; Reyburn et al., 2016; Sozo et al., 2015; Wang et al., 2014; Yee et al., 2016). In some instances, the lack of model standardisation might have resulted in opposite findings when evaluating the same candidate therapeutic intervention (Silva et al., 2015). These concerns might suggest the benefits of a standardised model of BPD.

Recent developments in the stereological analysis of tissue and organ structure have provided a tool that can be used to study pathological changes to the architecture of a target organ – even of a very small magnitude – with extraordinarily high precision. Similarly, these design-based stereology approaches can also be used to evaluate the efficacy of candidate therapeutic interventions that can correct pathological perturbations to organ structure. To this end, specific guidelines exist for the use of design-based stereology to study lung structure (Hsia et al., 2010), and the design-based stereology approach has been refined by the authors for the study of normal and aberrant lung development in the newborn mouse (Madurga et al., 2015, 2014; Mižíková et al., 2015).

Here, we report the use of design-based stereology to systematically explore the impact of oxygen on postnatal mouse lung development, considering both the level of oxygen in the inspired air, and the window of oxygen exposure. As in all organs, the structure of the lung is intimately related to lung function (Hsia et al., 2016), and lung structure in infants with BPD exhibit two pathological hallmarks: disturbance to the gas exchange structure (cumulatively reflected by a change in total number of alveoli, alveolar density and gas exchange surface area), as well as thickening of the alveolar septal walls. Both lung structural elements of BPD are recapitulated in the continuous exposure to 85% O_2_ for the first 14 days of life.

We have selected P14 as the end-point for our studies as P14 is the time-point at which the bulk of secondary septation is largely completed, and as such, even subtle perturbations to secondary septation will be suitably amplified and (relatively) easy to detect at P14. Given that secondary septation is broadly thought to extend at least to P10, P14 seemed to be a suitable time point. Furthermore, reference to Silva et al. (2015) revealed that P14 is the most commonly used end point for hyperoxia-based studies. As such, this is the best time point to terminate the studies presented here, to facilitate comparisons with the work of others. Our data have revealed some interesting effects of oxygen exposure protocols on the postnatal development of the mouse lung. Continuous exposure to as little as 40% O_2_ over the first 14 days of postnatal life did impact postnatal lung development, with continuous exposure to 40% O_2_, 60% O_2_, and 85% O_2_ having an impact on the number of alveoli (Fig. 2M), alveolar density (Fig. 2N), and gas exchange surface area (Fig. 2O), whereas only continuous exposure to 85% O_2_ impacts mean septal wall thickness (Fig. 2Q). In general, the impact of 40% O_2_ and 60% O_2_ were largely comparable considering the effect of oxygen exposure on number of alveoli and mean septal wall thickness. Some unexpected observations were also made. Notably, a decreasing O_2_ gradient from 85% O_2_ at P1 to 21% O_2_ at P14 was without any impact on number of alveoli (Fig. 2M) or mean septal wall thickness (Fig. 2Q), although the cumulative oxygen exposure exceeded that of the continuous 40% O_2_ group where both number of alveoli and mean septal wall thickness were impacted. It is speculated that after birth the immediate and dramatic (85% O_2_) oxygen toxicity recruited anti-oxidant lung protective pathways, which were progressively titrated down over time, commensurate with progressively decreasing O_2_ levels, thus maintaining the lung protective strategies at levels that were protective, but never damaging. Along similar lines, oscillating on a 24 h:24 h cycle between 85% O_2_ and 40% O_2_ also represented a cumulative oxygen exposure that was greater than the continuous 40% O_2_ protocol; however, the degree of damage to the lung was comparable, perhaps also indicating the recruitment and de-recruitment of lung protective strategies by sudden exposure to dramatic changes in inspired O_2_ levels.

The data presented here suggest that exposure of newborn mouse pups either to (1) 85% O_2_ from P1 to P7, followed by seven days of 21% O_2_ (Fig. 3E,K) or (2) to 85% O_2_ from P1 to P14 (Fig. 3F,L) generated the most dramatic impact on the lung architecture. Both protocols recapitulated the two key pathological hallmarks of BPD: blunted secondary septation (revealed by a decreased alveolar number) and an increased mean septal wall thickness; with a more pronounced effect on alveolar number (Fig. 3M), alveolar density (Fig. 3N), and MLI (Fig. 3P) in the P1-P14 85% O_2_-exposure group, suggesting that these two protocols best model BPD in mice. Although the continuous exposure to 85% O_2_ from P1 to P14 is a constant injurious insult, the impact of exposure to 85% O_2_ from P1 to P7, followed by 21% O_2_ between P7 and P14 is harder to define, as the latter protocol represents a period of injury, followed by a period where the lung might repair itself during room-air exposure. This might account for the less severe damage noted in the P1-P7 85% O_2_ group. Alternatively, lungs in this group might simply have been less damaged as a result of the reduced time frame of exposure to the injurious insult. Although the degree of damage to the developing lung in these two groups is largely comparable, the continuous exposure protocol (P1-P14) is proposed as the method of choice, as the lung repair mechanisms that might be engaged after return to 21% O_2_ in the P1-P7 exposure group represent confounding variables that are not present in the continuous exposure protocol (P1-P14), although it might be argued that that ‘oxygen recovery’ period is of translational significance. This ‘repair phase’ confounding variable might make data interpretation difficult, either when dissecting pathogenic pathways modulated by oxygen injury or when investigating the efficacy of a candidate drug to limit damage from oxygen injury. Of additional interest is the long-term sequelae of hyperoxia exposure in the neonatal period, which remains of interest to model as alveolarisation in humans continues into the teen years at least. However, the persistence of alveolarisation defects beyond P14 in mice was not considered in this study.

The P5.5 time point is believed to represent the peak of secondary septation (Herriges and Morrisey, 2014; Hogan et al., 2014), which is the driver of alveologenesis. Therefore, mouse pups were also exposed to 85% O_2_ over the period P4-P7 (Fig. 3D,J), believed to be a critical window of secondary septation. Mouse pups were also exposed to 85% O_2_ over the preceding period, P1-P3 (Fig. 3C,I). For both number of alveoli (Fig. 3M) and the mean septal wall thickness (Fig. 3Q), a dramatic impact was noted for the P4-P7 exposure periods, but not for the P1-P3 exposure period. These data might confirm that the period P4-P7 is a critical window of lung development that might be severely impacted by hyperoxia exposure; however, it is also noteworthy that the cumulative oxygen dose for the period P4-P7 is greater (as this period is 24 h longer) than the P1-P3 period.

It is important to note that the only readout used in this study was a change in lung structure, where interventions were examined for the ability to drive changes to the total number of alveoli in the lung and in the thickness of the alveolar septal wall. In selected instances, for example, exposure of newborn mouse lungs to hyperoxia over the first three days of life, no impact of an oxygen-exposure protocol on lung structure was noted (Fig. 1B, Fig. 3C,I). However, it might well be that other important changes do occur in the lung that are of physiological and pathological relevance, but which are not evident from an examination of the lung structure alone. Indeed, exposure of newborn mice to hyperoxia over the first three days of postnatal life has a dramatic impact on the airway hyper-responsiveness in the long-term (at days 55-77) in adult mice (Regal et al., 2014). Similarly, in the same model, an impact of early exposure to hyperoxia over the first three days of postnatal life was also documented to de-regulate the expression of epithelial (*Sftpc*, *Abca3*, *Pdpn*, *Aqp5*) as well as endothelial (*Pecam*) genes (Yee et al., 2014). Additionally, after slightly longer exposures (for the first four days of life) to hyperoxia, persistent changes in natural killer cell responses to influenza virus infection were observed in adult life (Reilly et al., 2015). It should be noted that these three studies employed 100% O_2_ as an injurious stimulus, but these reports do indicate that although alterations to lung structure were not noted after hyperoxia exposure for the first three days of postnatal life in our standardised BPD model presented here, other physiological changes clearly do occur in the lung.

A key concern in the use of experimental animal models of human disease is that often the most injurious stimulus is employed in an effort to obtain a clearly evident pathological change in a parameter of interest. In these instances, changes are dramatic and parameters are better clustered, yielding a more favourable statistical comparison with controls. In the case of the hyperoxia-based mouse model of BPD, the continuous exposure of newborn mouse pups to 85% O_2_ from P1 to P14 yielded the most pronounced impact on the gas exchange structure (cumulatively reflected by a change in total number of alveoli, alveolar density and gas exchange surface area; Fig. 2M-O) as well as disturbances to alveolar septal wall thickness (Fig. 2Q). However, one danger of this severe model is that a highly injurious stimulus might exert such a damaging effect that the positive (but moderate or weak) impact of a candidate intervention might go unnoticed. Therefore, this idea was tested by providing nutritional supplementation to newborn mouse pups (in the form of cottonseed oil) over the course of oxygen exposure between P1 and P14, at both 60% O_2_ and 85% O_2_, and examined the magnitude of improvement in lung structure in both oxygen-exposure protocols. Indeed, a beneficial impact of cottonseed oil administration was noted in the 60% O_2_ exposure protocol, but not in the 85% O_2_ exposure protocol. Thus, if the 85% O_2_ model alone had been employed, the potential utility of cottonseed oil as an intervention in BPD would have been missed. The selection of the oxygen exposure is, therefore, crucially important; and these observations underscore the idea that if an experimental intervention proves unsuccessful in severe models of disease, a less severe model might nevertheless highlight the potential utility of the intervention. Further to this, however, the severe (85% O_2_) BPD model is the only model that recapitulates both the disturbances to the gas exchange structure (cumulatively reflected by a change in total number of alveoli, alveolar density and gas exchange surface area; Fig. 2M-P,R) as well as disturbances to alveolar septal wall thickness that are seen in human disease (Fig. 2Q). Although an effect of cottonseed oil administration on alveoli number in the 85% O_2_ model was missed, a pronounced positive impact of cottonseed oil on alveolar septal wall thickness was still noted in the 85% O_2_ model, where cottonseed oil application normalised the alveolar wall thickness (Fig. 4K,L, versus Fig. 4I,J; Fig. 4Q). Taken together, these data reveal that the oxygen exposure protocol requires tailoring and broad consideration when assessing the potential utility of an intervention intended to drive postnatal lung maturation in an experimental animal model of BPD. It is the recommendation of the authors to employ 85% O_2_ to generate an impact of hyperoxia on both the number of alveoli and the mean septal wall thickness. However, when an experimental intervention fails to blunt the impact of 85% O_2_ on these two parameters of lung structure, it is recommended to evaluate the same intervention using 60% O_2_ as an injurious stimulus. Keeping animal welfare in mind (Curzer et al., 2016), this model represents the best compromise between minimising the number of experimental animals employed and the level of stress to which the animal is subjected by keeping the injurious insult as mild as possible, while still retaining the translational viability of the animal model.

This report exclusively addresses the use of hyperoxia as an injurious stimulus in the arrested lung development associated with hyperoxia exposure, as a model for BPD. Clinically, hyperoxia is one of many contributors to BPD, with additional important disease modifiers including, *inter alia* baro- and volu-trauma from mechanical ventilation (Greenough et al., 2008), and the background of infection (Balany and Bhandari, 2015). Future efforts to further optimise the mouse (and other) models of BPD should address hyperoxia in combination with mechanical ventilation and/or infection.

## MATERIALS AND METHODS

### Approvals for studies with experimental animals

All animal procedures were approved by the local authorities, the Regierungspräsidium Darmstadt (approval numbers B2/344, B2/1051, and B2/1108).

### Mouse models of bronchopulmonary dysplasia

The normobaric hyperoxia-based model of BPD in mice was conducted essentially as described previously, with the modifications outlined below (Alejandre-Alcázar et al., 2007; Madurga et al., 2014; Mižíková et al., 2015). Newborn C57Bl/6 mice (*Mus musculus* Linnaeus) were randomised to equal-sized litters (average seven mice per litter), and placed into either a normoxic or hyperoxic environment within two hours of birth. The hyperoxia-exposure protocols are illustrated in Fig. 1A,B. For the 40% O_2_, 60% O_2_, and 85% O_2_ oxygen exposure protocols, mouse pups were exposed to the appropriate oxygen concentration starting on the day of birth (P1), continuously up to and including P14. Two additional oxygen level protocols were also performed: (1) a decreasing gradient of O_2_ from 85% on P1 to 21% on P14 (a reduction in oxygen concentration of 5% per day); and (2) an oscillation between 85% O_2_ and 40% O_2_, for a 24 h period, on a 24 h:24 h oscillation cycle.

To determine the necessary window of oxygen exposure over the first 14 days of life, newborn mouse pups were exposed to 85% O_2_ for discrete ‘windows’, which included: (1) the first 24 h of life (P1), (2) the first three days of life, starting at P1, up to and including P3; (3) starting at the beginning of P4 and continuing to (and including) P7; (4) the first seven days of life, starting at P1, and continuing to (and including) P7; and (5) the entire first 14 days of life, ending with (and including) P14. All experiments were terminated at P14.

For all oxygen-exposure protocols, nursing dams were rotated every 24 h, to ensure at least one 24 h period of 21% O_2_ every 2 days. This addresses the oxygen toxicity issues in adult mice, which are highly susceptible to prolonged periods of hyperoxia. Nursing dams received food *ad libitum*. Mice were maintained in a 12 h:12 h dark/light cycle. All pups were euthanised at the end of P14 with an overdose of pentobarbital (500 mg/kg, intraperitoneal; Euthoadorm, CP-Pharma, Burgdorf, Germany), followed by thoracotomy, then by lung extraction and processing for design-based stereology (Madurga et al., 2014; Mižíková et al., 2015).

### Design-based stereology

All methods employed for the analysis of lung structure were based on American Thoracic Society and European Respiratory Society recommendations for quantitative assessment of lung structure (Hsia et al., 2010). The protocol employed for the design-based stereological analysis of neonatal mouse lungs has been described in the detail previously (Madurga et al., 2015, 2014; Mižíková et al., 2015), based on state-of-the-art methodology (Mühlfeld et al., 2015, 2013; Mühlfeld and Ochs, 2014, 2013; Ochs and Mühlfeld, 2013; Schneider and Ochs, 2013; Schneider and Ochs, 2014). Briefly, mouse lungs were instillation-fixed through a tracheal cannula at a hydrostatic pressure of 20 cm H_2_O with 1.5% (w/v) paraformaldehyde (Sigma, Darmstadt, Germany; P6148), 1.5% (w/v) glutaraldehyde (Serva, Heidelberg; 23116.02) in 150 mM HEPES (Sigma; H0887), pH 7.4, for 24 h at 4°C, after which lung tissue blocks were collected according to systematic uniform random sampling for stereological analysis (Schneider and Ochs, 2014).

Lungs were embedded *in toto* in 2% (w/v) agar (Sigma; 05039) and cut into 3 mm sections. The total volume of the lungs was measured by Cavalieri's principle (Madurga et al., 2015, 2014; Mižíková et al., 2015). Whole lungs were treated with sodium cacodylate (Serva; 15540.03), osmium tetroxide (Roth, Karlsruhe, Germany; 8371.3), and uranyl acetate (Serva; 77870.01) and embedded in glycol methacrylate (Technovit 7100; Heareus Kulzer, Hanau, Germany; 64709003). For the determination of alveoli number, each tissue block was cut into sections of 2 µm, and every first and third section of a consecutive series of sections throughout the block was stained with Richardson's stain. For all other parameters, every tenth section of a consecutive series throughout the block was similarly prepared (four sections per block were selected).

All slides were scanned using a NanoZoomer-XR C12000 Digital slide scanner (Hamamatsu, Herrsching am Ammersee, Germany). Analyses were performed using the Visiopharm NewCast computer-assisted stereology system (Visiopharm, Hoersholm, Denmark). Parameters analysed included the mean linear intercept (MLI), alveolar septal wall thickness, total surface area, as well as alveolar number and alveolar density, as described previously (Madurga et al., 2015, 2014; Mižíková et al., 2015). Intrinsic to this analysis is the separate scoring of parenchymal elements, which are discriminated from vessels and airways. A total of ∼40 tissue sections were evaluated per animal, for all parameters, except the determination of alveolar number, in which case a total of 10 sections per animal were evaluated. In each case, 2-5% of each section was analysed. The coefficient of error (CE), the coefficient of variation (CV), as well as the squared ratio between both (CE^2^/CV^2^) were measured for each stereological parameter (Tables 1,2,3), and the quotient threshold was set at 0.5 to validate the precision of the measurements.

### Therapeutic intervention with generic parenteral nutrition

Cottonseed oil (Sigma-Aldrich, Darmstadt, Germany; C7767) was applied by daily intraperitoneal injection to pups, where the cottonseed oil dose was de-escalated over a range starting at 20 ml kg^−1^ day^−1^ (P1, P2, P3), followed by 15 ml kg^−1^ day^−1^ (P4, P5, P6), then by 10 ml kg^−1^ day^−1^ (P7, P8, P9) and finally to 5 ml kg^−1^ day^−1^ (P10, P11, P12, and P13), to avoid the injection of a large oil bolus. The experiment was terminated at P14. The cottonseed oil application was undertaken in the BPD model using either continuous exposure to 60% O_2_ (FiO_2_ 0.60) or 85% O_2_ (FiO_2_ 0.85). The application of cottonseed oil was in analogy with generic oil-based nutritional supplementation that is provided to preterm infants with or at risk for BPD in a neonatal intensive care setting (Beken et al., 2014).

### Sex genotyping of mice

Sex determination of mouse pups was undertaken exactly as described previously (Lambert et al., 2000). Essentially, genomic DNA was isolated from tail biopsies, and screened by polymerase chain reaction using forward primer 5′-TGGGACTGGTGACAATTGTC-3′ and reverse primer 5′-GAGTACAGGTGTGCAGCTCT-3′ to detect the male-specific *Sry* locus; together with forward primer 5′-GGGACTCCAAGCTTCAATCA-3′ and reverse primer 5′-TGGAGGAGGAAGAAAAGCAA-3′ to detect the *Il3* gene present in both the male and female sex. Amplicons were resolved on a 1.5% (w/v) agarose gel, and visualised by ethidium bromide staining.

### Statistical analysis

All data are presented as mean±s.d. Differences between groups were evaluated by one-way ANOVA with Tukey's post hoc test for all experiments. *P*-values <0.05 were regarded as significant. All statistical analyses were performed with GraphPad Prism 6.0. The presence of statistical outliers was tested by Grubbs' test, and none were found. All data sets are small for proper determination of normal distribution; however, a corrected Anderson–Darling statistic demonstrated normal distribution of data sets.
